# Papillary Thyroid Carcinoma Landscape and Its Immunological Link With Hashimoto Thyroiditis at Single-Cell Resolution

**DOI:** 10.3389/fcell.2021.758339

**Published:** 2021-11-05

**Authors:** Jun Pan, Fang Ye, Chengxuan Yu, Qinsheng Zhu, Jiaqi Li, Yaohui Zhang, Hedi Tian, Yunjin Yao, Minjie Zhu, Yibin Shen, Feng Zhu, Yingying Wang, Xinhui Zhou, Guoji Guo, Yijun Wu

**Affiliations:** ^1^Department of Thyroid Surgery, The First Affiliated Hospital, School of Medicine, Zhejiang University, Hangzhou, China; ^2^Center for Stem Cell and Regenerative Medicine, The First Affiliated Hospital, Zhejiang University School of Medicine, Hangzhou, China; ^3^Surgical Department, Hangzhou Third Hospital, Hangzhou, China; ^4^Kidney Disease Center, The First Affiliated Hospital, School of Medicine, Zhejiang University, Hangzhou, China; ^5^Department of Gynecology, The First Affiliated Hospital, School of Medicine, Zhejiang University, Hangzhou, China; ^6^Liangzhu Laboratory, Zhejiang University Medical Center, Hangzhou, China; ^7^Zhejiang Provincial Key Laboratory for Tissue Engineering and Regenerative Medicine, Dr. Li Dak Sum & Yip Yio Chin Center for Stem Cell and Regenerative Medicine, Hangzhou, China; ^8^Institute of Hematology, Zhejiang University, Hangzhou, China; ^9^Stem Cell Institute, Zhejiang University, Hangzhou, China

**Keywords:** papillary thyroid cancer, Hashimoto’s thyroiditis, single-cell RNA sequencing, tumor B-cell infiltration, immunological link

## Abstract

The tumor microenvironment heterogeneity of papillary thyroid cancer (PTC) is poorly characterized. The relationship between PTC and Hashimoto thyroiditis (HT) is also in doubt. Here, we used single-cell RNA sequencing to map the transcriptome landscape of PTC from eight PTC patients, of which three were concurrent with HT. Predicted copy number variation in epithelial cells and mesenchymal cells revealed the distinct molecular signatures of carcinoma cells. Carcinoma cells demonstrated intertumoral heterogeneity based on *BRAF* V600E mutation or lymph node metastasis, and some altered genes were identified to be correlated with disease-free survival in The Cancer Genome Atlas datasets. In addition, transcription factor regulons of follicular epithelial cells unveil the different transcription activation state in PTC patients with or without concurrent HT. The immune cells in tumors exhibited distinct transcriptional states, and the presence of tumor-infiltrating B lymphocytes was predominantly linked to concurrent HT origin. Trajectory analysis of B cells and plasma cells suggested their migration potential from HT adjacent tissues to tumor tissues. Furthermore, we revealed diverse ligand–receptor pairs between non-immune cells, infiltrating myeloid cells, and lymphocytes. Our results provided a single-cell landscape of human PTC. These data would deepen the understanding of PTC, as well as the immunological link between PTC and HT.

## Introduction

Thyroid cancer is the most common endocrine malignancy worldwide, with significant increases in incidence around the world over the past three decades (Pellegriti et al., 2013). The global incidence rate in women is 10.2 per 100,000, and that in men is 3.1 per 100,000. This disease represents 5.1% of all estimated female cancer burden (Bray et al., 2018). Papillary thyroid cancer (PTC) is the most prevalent subtype of thyroid cancer, accounting for 70 to 85.9% of the incidence of all thyroid cancers (Kwak et al., 2010). Although PTC is generally indolent and shows a favorable prognosis, some metastasized lesions are not treatable with radioactive iodine or surgery therapy.

With the advances in genomic and transcriptome sequencing technologies, some potential marker genes for PTC diagnosis and progression have been identified. *BRAF/RAS* gene alteration matched treatment represents the gene-targeted therapy. Besides, gene expression–based molecular subtyping of PTC could be potentially used as a prognostic tool with important clinical relevance. *BRAF* V600E mutation has the strong predictive power for the diagnosis and aggressive pathological results of PTC (Kebebew et al., 2007; Xing et al., 2014; Wang et al., 2016). However, conventional genomic and gene expression profiling is applied to characterize a bulk tumor and ignore the intertumoral heterogeneity. PTC is a highly heterogeneous tumor composed of diverse populations of lymphocytes and myeloid cells that affect tumor initiation, progression, and treatment resistance. Some studies have reported a correlation between positive or negative clinical outcomes in thyroid cancer patients and tumor-infiltrating immune cells (Gooden et al., 2011; Gentles et al., 2015). Myeloid-derived suppressor cells are elevated in cancer patients, where they show a strong immunosuppressive potential, and are associated with a poor prognosis (Gabrilovich and Nagaraj, 2009). The frequency of tumor-associated macrophages (TAMs) changes in the distinct subtypes of thyroid cancer (Jung et al., 2015). Natural killer (NK) cells also play a central role in thyroid cancer immunosurveillance (Lee and Sunwoo, 2019). The density of lymphocytes has been reported to be correlated with improved overall survival and lower recurrences of PTC (Kuo et al., 2017). Thus, it is important to characterize tumor cells as well as immune cells to clarify their property in PTC.

Papillary thyroid cancer displayed a slow growth and lymphatic spread with rarely distant metastasis, which might be due to the frequent presence of lymphatic infiltration into tumor sites. It is reported that 18.9 to 23.2% of the PTC is concurrent with Hashimoto thyroiditis (HT), and PTC patients with synchronous appearance of HT had better prognosis compared with those without HT (Ahn et al., 2011; Konturek et al., 2013; Lee et al., 2013; Zhang et al., 2014; Resende de Paiva et al., 2017). In PTC patients with or without concurrent HT, whether tumor-infiltration lymphocytes are attracted by an antitumor immune response or influenced by a preexisting autoimmune process remains unknown. At least, an immunological link between PTC and HT could not be excluded, but the role of HT in shaping the PTC immune milieu is still unclear.

Since the first single-cell RNA sequencing (scRNA-seq) technique was published in 2009 (Tang et al., 2009), scRNA-seq has gradually become a cutting-edge method to resolve intratumor heterogeneity. Single-cell transcriptomes provided new insights into cell–cell interactions in multiple types of cancer (Zhang et al., 2019; Davis et al., 2020; Wieland et al., 2020). Some molecular studies reported the advances in follicular thyroid carcinoma and medullary thyroid carcinoma (Ceolin et al., 2012; Mohammadi and Hedayati, 2017; Borowczyk et al., 2019; Oczko-Wojciechowska et al., 2020). A recent scRNA-seq study made a comparison of tumor microenvironment (TME) in PTC between genders (Peng et al., 2021). Another most recent study delineated dedifferentiation process of anaplastic thyroid cancer and PTC at a single-cell level (Luo et al., 2021). In this study, took the advantage of Microwell-seq, a low-cost and high-throughput scRNA-seq platform that has been reported in our previous studies (Han et al., 2018, 2020), we constructed the single-cell transcriptome landscape of human papillary thyroid carcinoma. The systemic single-cell transcriptome data provided novel insights to understand the TME of PTC. We explored cell composition, functional states, cellular interactions in PTC tumors, and adjacent thyroid tissues (with HT). Specifically, previously uncharacterized HT, which PTC is often concurrent with, was included in this study to investigate the dynamic relationship of immunocytes. Taken together, the precise characterization of PTC and its microenvironment, in combination with the immunological crosstalk with HT, facilitates in-depth understanding of the PTC molecular characteristics. These results would also help in the identification of potential molecular targets for PTC diagnosis and treatment.

## Materials and Methods

### Sample Collection

This study was approved by the Clinical Research Ethics Committee of the First Affiliated Hospital, School of Medicine, Zhejiang University (IIT consent: no. 700,2020), and carried out in accordance with the principles of the Declaration of Helsinki. Eight patients, including three concurrent with HT, diagnosed with PTC were recruited in our study, and all the patients signed the informed written consent for each subject and agreed to donate the specimens. A total of 10 fresh tissue samples (five samples from PTC patients without concurrent HT, three samples from PTC patients with concurrent HT, and two paired adjacent tissues of two PTC patients with concurrent HT) were collected from Chinese PTC patients undergoing thyroidectomy at the Department of Thyroid Surgery of the hospital. Paired adjacent tissues were collected by curettage at the same time as tumor tissue collection. The patients did not receive any other forms of therapy. Diagnosis of PTC and HT cases was histologically confirmed by two independent pathologists, and all of the tumor tissues were assessed by hematoxylin-eosin staining.

### Single-Cell Suspension Preparation

Fresh and sterile tumor tissue fragments were initially divided into segments, after two washings with 1 × phosphate-buffered saline, the tumor pieces were dissociated into single-cell suspensions through Human Tumor Dissociation Kit (Miltenyi Biotec GmbH, Bergisch Gladbach, Germany) according to the kit manuals. Digested tumor pieces were teased through a 40-μm sieve. Then, the dissociated single cells were centrifuged, and cell pellets were resuspended in PRIM1640 (Thermo Fisher Scientific) plus 0.04% bovine serum albumin (Sigma–Aldrich). Viability was confirmed to be >90% in all samples *via* trypan blue (Thermo Fisher Scientific) staining, and the cell suspensions were kept on ice for the scRNA-seq.

### Single-Cell RNA Library Preparation and Sequencing

Standard Microwell-seq protocol was performed to treat single-cell suspensions from different samples. In brief, single-cell suspensions and barcode beads were loaded on agarose Microwell array. Beads and cells were trapped in separated Microwells. Transcripts from lysed cell were captured by barcode oligodT bead. Beads were collected in a 1.5-mL tube to do template switch, reverse transcription, exonuclease I treatment, and cDNA amplification. Purified cDNA libraries were tagmented using a customized transposase to enrich 3’ ends of transcripts (TruePrep DNA Library Prep Kit V2 for Illumina, Vazyme, cat. no. TD513), Libraries were sequenced on Illumina Hiseq Xten (PE150 mode) by Novogene Co., Ltd., Beijing, China.

### Single-Cell RNA Sequencing Data Processing

Drop-seq core computational tool (version 1.12) was used to preprocess the Microwell-seq raw data. As described in Drop-seq computational cookbook^1^. Online R packages of data preprocessing and detailed parameters are available at Github^2^. Filtered reads were used to extract cellular barcode and unique molecular identifier (UMI). We discarded the paired reads if the quality of any base in the barcode was below 10. STAR (version 2.5.2a) with default parameters was used for mapping. Reads were aligned to the *Homo sapiens* GRCh38 reference genome. All multialigned reads were removed, and GTF annotation files from GENCODE were used to tag aligned reads. For UMI count, molecular barcodes with one edit distance were merged to one within a gene. We excluded cells in which there were fewer than 500 UMIs. All the R packages were loaded in R (version 3.6.3), and plots were mapped using R package ggplot2 (version 3.3.5).

### Cell Type Clustering and Annotation

After obtaining the digital gene expression data matrix, we used R package Seurat (version 3) for dimension reduction. Preprocessed cells and genes expressed in more than three cells were selected for further clustering and differential gene expression analysis^3^. Filtered data were ln (CPM/100 + 1) transformed. We selected the top 2,000 highly variable genes and top 20 principal components for clustering. Default Wilcoxon rank sum test was used by running FindAllMarkers function in Seurat to find differentially expressed markers in each cluster (min.pct = 0.25, logfc.threshold = 0.25). Uniform Manifold Approximation and Projection (UMAP) was adopted for cluster visualization. We compared known markers with human cell landscape database^4^ to achieve cell type annotation.

### The Cancer Genome Atlas THCA Dataset Validation

#### Data Processing and Single-Cell Subsets Correlation Calculation

Transcriptome data from The Cancer Genome Atlas (TCGA) THCA datasets were downloaded from UCSC XENA^5^. Genes with average logFC > 1 were used as marker genes of each cell type. We used Spearman correlation analysis to estimate correlation between immune cell types.

#### Expression Analyzing of Selected Genes

A total of 568 cases with gene expression data (HTSeq-counts and HTSeq-FPKM) in THCA projects were collected from TCGA. Among them, those with clinical information were included. Then, counts and FPKM data were transformed into TPM for the following analyses. The TPM data for 500 patients were used for further analyses. All statistical analysis and plots in this validation part were produced using R (v4.0.3). Wilcoxon rank sum test and signed rank test were used to analyze the expression of selected genes in PTC samples and PTC combined with HT samples.

#### Survival Analysis of Selected Genes

To determine the best cutoff value of selected genes to predict disease recurrence in PTC patients, the X-tile software was used. Then, the 500 samples were divided into two groups, high-expression group and low-expression group according to the best cutoff value. Next, Kaplan–Meier method was applied to conduct the survival analysis and plot the survival curves of selected genes. In all tests, *p* < 0.05 was considered statistically significant.

#### Tumor-Infiltrating Immune Cell Profile

CIBERSORT computational method was applied for estimating the tumor-infiltrating immune cells abundance profile in all 500 samples. The profile of 21 types of immune cells was displayed by boxplot.

### RNA Velocity Analysis

Velocyte (version 0.17) was used to calculate RNA velocity of B cell in samples from PTC patients with HT and adjacent tissue samples from PTC patients with HT. The rates of transcriptional changes of each cell were estimated using the ration of spliced and unspliced reads^6^ with default parameters. The plot was visualized with UMAP embedding. The differentiation start and end points were estimated using a Markov process with default parameters.

### Single-Cell Pseudotime Trajectory Analysis

We selected differentially expressed genes (DEGs) of immune cells (B cells, plasma cells). We use monocle2 R package (version 2.4.0) to treat genes expressed in at least three cells in single-cell data. Default settings of DEGs were adopted to construct pseudotime trajectory and heatmap.

### Receptor–Ligand Pairing Analysis

We used CellPhoneDB (version 2.1.3) for the analysis of potential receptor–ligand pairings. We aggregated the gene expression levels of all clusters in samples from PTC patients with HT and adjacent tissue from PTC patients with HT. Receptors and ligands expressed in more than 10% of the cells in each cluster were considered. The cutoff was set with the mean expression greater than 0.05 and *p* values smaller than 0.05. We used the sum of the number of receptor–ligand pairs in each cell–cell pairing to indicate the strength of the cell–cell interactions. The interaction network was visualized using Cytoscape (version 3.7.0) and ggplot2 (version 3.3.5).

### Metric Learning

Metric learning is a machine learning model for the purpose of constructing distance metrics from supervised data, and the constructed distance metrics could be used for further clustering, classification, and information retrieval. Here, we used PyTorch Metric Learning tool (version 0.9.90)^7^ for designing a binary classifier and applied it to predict the source of follicular epithelial cells (PTC patients with concurrent HT or PTC patients without concurrent HT; by sampling triplets with default parameters). Training sets and validation sets were randomly selected and generated from our scRNA-seq data. To extract and explore genes that have important contributions to classification, we used Captum (version 0.4.0)^8^ for model interpretation and understanding. Finally, an R package pheatmap was applied to visualize the gene importance in prediction of the source of follicular epithelial cells.

### Immunohistochemistry

Immunohistochemistry (IHC) staining was performed on 4-μm-thick, formalin-fixed, paraffin-embedded sections using an Opal multiplex IHC system (NEL811001KT, PerkinElmer) according to the manufacturer’s instructions. For IHC, tissue sections were subjected to antigen retrieval in an induction cooker for 25 min in EDTA buffer (pH 9.0). Followed by treatment with goat serum at 37°C for 40 min, tissue sections were incubated with the following antibodies: TG (ab151539), FOS (ab184938), JUN (ab178858), TFF3 (ab109104), CD55 (ab133684), CCDC80 (ab224050), CD79A/B (ab79414), and CD3D (ab109531) at 4°C overnight. All antibodies were from Abcam (Cambridge, MA, United States). Images were recorded with Metamorph software v7.5.6.0 (Molecular Device) on an Olympus IX81 inverted microscope. The images were evaluated by two independent pathologists who were blinded to the patients’ clinical information.

## Results

### Single-Cell Profiling of Papillary Thyroid Cancer

**FIGURE 1:**
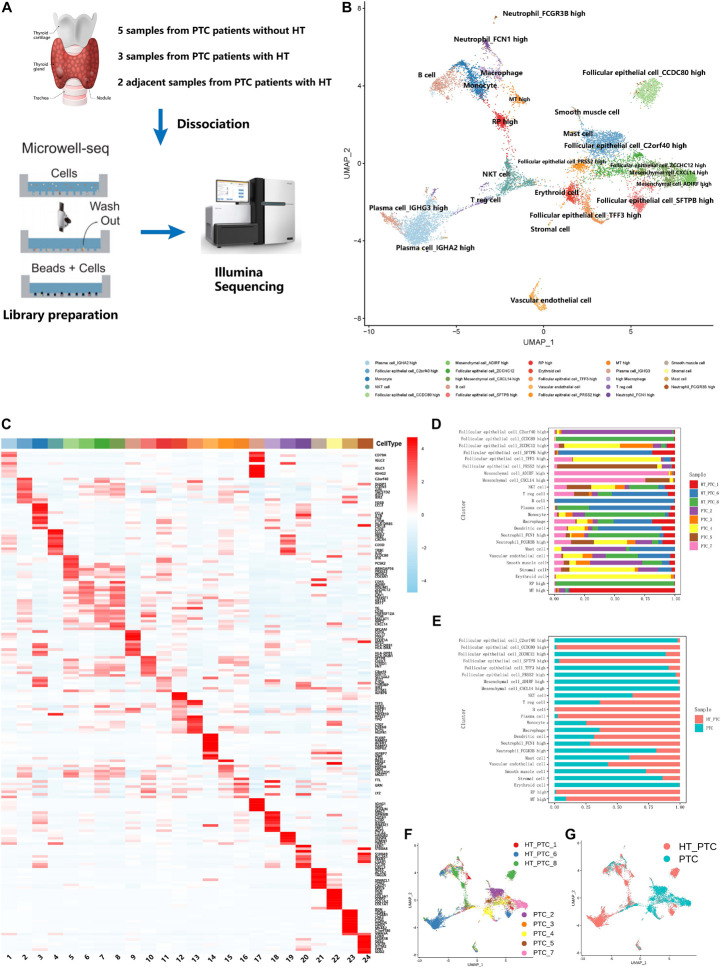
Single-cell transcriptome landscape of papillary thyroid cancer. **(A)** Workflow of sample dissociation, library preparation, and sequencing. **(B)** UMAP plot of subclusters in PTC cell landscape. **(C)** Heatmap of specific marker genes of paired clusters in **(B)**. **(D)** The fractions of samples in each cell subcluster. **(E)** The fractions of sample types in each cell subcluster. **(F)** UMAP plot of cells colored by PTC patients. PTC, samples from PTC patients without concurrent HT; HT_PTC, samples from PTC patients with concurrent HT. **(G)** UMAP plot of single cells colored by PTC types.

To construct the single-cell landscape of PTC, we applied Microwell-seq to study carcinoma and non-carcinoma microenvironment cells of tumor samples from eight PTC patients diagnosed histologically, with three patients concurrent with HT. Clinical information of all patients is shown in Table 1. Diagnosis of all of the PTC patients [including lymph node metastasis (LNM), HT, and *BRAF* V600E mutation] was histologically confirmed by two independent pathologists. Tumor samples from PTC patients and adjacent tissue samples from PTC patients with concurrent HT were digested into single cells for standard workflow of Microwell-seq (Figure 1A). After removing low-quality cells, we generated 14,252 cells in eight tumor samples from PTC patients and 9,178 cells in two adjacent tissue samples (HT tissues) from PTC patients with concurrent HT for downstream clustering. The aggregated single-cell transcriptome landscape of eight tumor samples (five samples from PTC patients without concurrent HT, three samples from PTC patients with concurrent HT) was visualized by UMAP plot (Figure 1B). We annotated 24 major clusters based on canonical marker genes, including T cells, B cells, plasma cells, macrophages, dendritic cells (DCs), neutrophils, mast cells, endothelial cells, smooth muscle cells, mesenchymal cells, and follicular epithelial cells. Heatmap showed the differentially expressed marker genes of each cluster (Figure 1C). Sample proportion in each cell type is shown (Figures 1D,F). We kept the original heterogeneity of patients without using any batch remove algorithm. Notably, plasma cells specifically expressing immunoglobulin and XBP1 and B cells specifically expressing CD79A and MS4A1 were restricted in samples from PTC patients with concurrent HT (Figures 1E,G). Besides B cells and plasma cells, PTC patients with concurrent HT and PTC patients without concurrent HT shared the most common immune cells. We identified six follicular epithelial cell subsets marked by TG, EPCAM, and malignant genes such as FOS and JUN. Those follicular epithelial cell subsets could also be distinguished by specific marker genes in each cluster. Most follicular epithelial cells_ZCCHC12 high, follicular epithelial cells_C2orf40 high, follicular epithelial cells_TFF3 high, and follicular epithelial cells_PRSS2 high were contributed by samples from PTC patients without concurrent HT (Figure 1E).

**TABLE 1:** Clinical characteristics of patients.

Patient	Gender	Tumor size(cm)	With LNM	With Hashimoto thyroiditis	*BRAF* V600E (mutation:+, wild type: –)	TSH (μIU/mL)^a^	T4 (nmol/L)^b^	T3 (nmol/L)^c^	FT (pmol/L)^d^	FT3 (pmol/L)^e^	TPOAB (IU/mL)^f^	TGAB (IU/mL)^g^
HT_PTC_1	Female	1.15 × 0.49	–	+	+	1.864	112.59	1.42	15.7	4.71	0.46	65.98
PTC_2	Female	1.4 × 1.0	+	–	+	0.553	98.25	1.2	16.78	3.56	0.81	2.08
PTC_3	Female	1.4 × 0.9	+	–	+	3.111	78.99	1.44	12.01	4.24	0.34	2.22
PTC_4	Male	0.7 × 0.4	–	–	–	2.077	88.42	1.68	12.96	4.83	<0.50	1.23
PTC_5	Female	1.1 × 0.8	+	–	+	1.581	81.5	1.53	12.73	4.4	<0.50	0.71
HT_PTC_6	Female	1.5 × 0.8	+	+	+	1.89	131.3	2.28	15.29	6.76	98.8	74.9
PTC_7	Male	1.4 × 1.0 × 0.8	+	–	+	0.794	102.7	1.71	12.22	5.41	<0.50	NA
HT_PTC_8	Female	1.8 × 1.1	+	+	–	0.763	100.6	1.46	12.46	4.49	100.57	490.71

*Abbreviations: ^a^Thyroid-stimulating hormone. ^b^Thyroxine. ^c^Triiodothyronine. ^d^Free thyroxine. ^e^Free triiodothyronine. ^f^Thyroid peroxidase antibody. ^g^Thyroglobulin antibody.*

### Intrinsic Parenchymal Cell Heterogeneity of Papillary Thyroid Cancer

**FIGURE 2:**
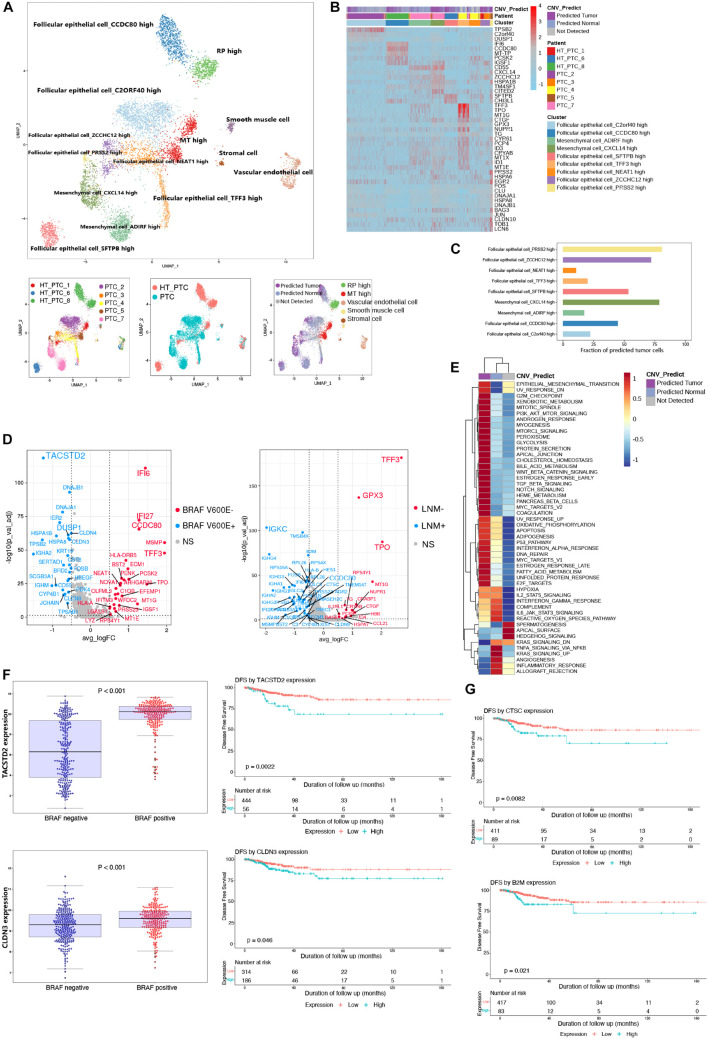
Reclustering of parenchymal cells and identification of malignant cells. **(A)** UMAP plot of parenchymal cell subclusters colored by cell types, patients, PTC types, and CNV prediction. **(B)** Heatmap shows the DEGs of clusters, patients, and CNV groups. PTC, samples from PTC patients without concurrent HT; HT_PTC, samples from PTC patients with concurrent HT. **(C)** Fractions of predicted tumor cells in each cell subcluster. **(D)** Volcano plots show DEGs of follicular epithelial cells based on *BRAF* V600E mutation and LNM. *BRAF* V600E^+^, highly expressed genes in patients with *BRAF* V600E mutation; *BRAF* V600E^–^, highly expressed genes in patients without *BRAF* V600E mutation; LNM^+^, highly expressed genes in patients with LNM; LNM-, highly expressed genes in patients without LNM; NS, genes with no significant expression patterns. **(E)** GSVA pathway enrichment of different group based on CNV level. **(F)** Expression level of TACSTD2 and CLDN3 between different mutation groups and DFS analysis in the TCGA-THCA cohort. **(G)** DFS analysis of CTSC and B2M in the TCGA-THCA cohort.

We merged parenchymal cell datasets (epithelial cells, stromal cells, mesenchymal cells, muscle cells, and endothelial cells) from eight patients (Figure 2A). Most clusters from PTC patients with or without concurrent HT were labeled by their original annotations. It was speculated that tumor cells and non-tumor cells were the origin of intratumoral heterogeneity due to the copy number variations (CNVs). Thus, we adopted CopyKAT (Gao et al., 2021) to evaluate the malignancy of follicular epithelial cells and mesenchymal cells based on genomic copy number estimation. Follicular epithelial cells_ ZCCHC12 high, mesenchymal cells_CXCL14 high, and follicular epithelial cells_PRSS2 high harbored highly CNV levels and contained most malignant cells (Figures 2A,C). Most predicted tumor cells showed gene amplification in chromosomes 14 and 15 (Supplementary Figure 1A). We then examined the expression patterns between predicted normal cells and predicted tumor cells (Figure 2B). Malignant mesenchymal cells exhibited high expression levels of CD55, and malignant epithelial cells exhibited high expression levels of TFF3. Gene set variation analysis of pathway demonstrated epithelial–mesenchymal transition (EMT), G2M checkpoint, E2F targets, MYC targets, and P53 pathway were specifically enriched in predicted tumor group with high CNV levels, whereas inflammatory response pathway was down-regulated (Figure 2E). These results unveiled an activated EMT state in tumor cell microenvironment of PTC, while immunogenicity of those tumor cells was inhibited.

As an important diagnostic and prognostic molecular marker, we detected the *BRAF* V600E mutation of all patients (Xing, 2010). The LNM was also regarded as an important prognostic factor. Follicular epithelial cells were grouped based on *BRAF* V600E mutation and LNM, respectively, (Figure 2D). DEGs in *BRAF* V600E mutation group and LNM group may associate with a poor disease-free survival (DFS) in PTC. Among single-cell DEGs in *BRAF* V600E mutation group, TACSTD2 and CLDN3 showed high expression levels in samples from TCGA THCA cohort (Figure 2F). TACSTD2 and CLDN3 were also significantly related to poor DFS in PTC patients. The expression levels of TACSTD2 was correlated with TNM *via* controlling up-regulation of MMP2 (Guan et al., 2017). The overexpression of CLDN3 was also considered to be correlated with thyroid cancer and other malignancies (Hewitt et al., 2006; Hess et al., 2011). In LNM-positive group, epithelial cells showed unique expression of CTSC and B2M that were related to poor DFS in PTC patients (Figure 2G; Wu et al., 2018).

### Reclustering of Follicular Epithelial Cells

**FIGURE 3:**
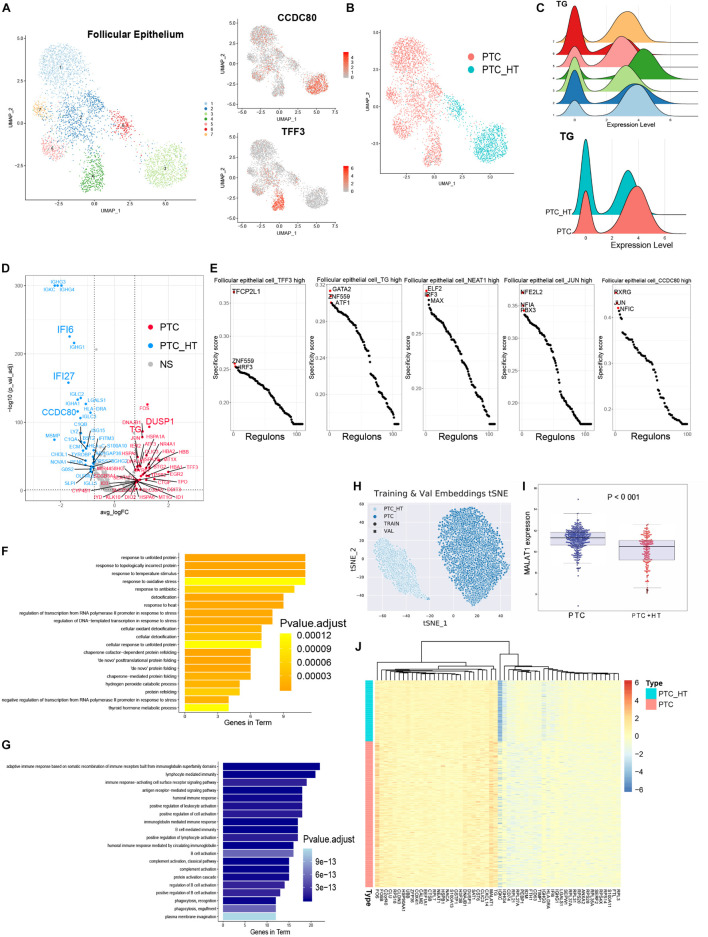
Identifying transcriptome signatures of follicular epithelial cells. **(A)** UMAP plot of follicular epithelial cell subclusters and FeaturePlot of marker genes in two types of PTC. **(B)** UMAP plot of follicular epithelial cells colored by PTC types. **(C)** Density plots of TG expression levels and cell number distribution in follicular epithelial cell subclusters. **(D)** Volcano plot shows DEGs of epithelial cells from two types of PTC. PTC, highly expressed genes in patients without concurrent HT; PTC_HT, highly expressed genes in patients with concurrent HT; NS, genes with no significant expression patterns. **(E)** Rank of the cell type specific regulons in follicular epithelial cell subclusters. **(F)** Gene ontology enrichment of the top 20 genes in follicular epithelial cells from PTC patients without concurrent HT. **(G)** Gene ontology enrichment of the top 20 genes in follicular epithelial cells from PTC patients with concurrent HT. **(H)** t-SNE plot of follicular epithelial cells clustering patterns using metric learning model. TRAIN, training set; VAL, validation set. **(I)** MALAT1 expression level in the TCGA-THCA cohort based on PTC types. PTC, PTC patients without concurrent HT; PTC + HT, PTC patients with concurrent HT. **(J)** Heatmap of genes contributed to the classification in metric learning model.

To further profile follicular epithelial cells from PTC patients, we performed unsupervised reclustering of all epithelial cells and generated seven clusters that could be annotated based on their original definition (Figure 3A and Supplementary Figure 2A). Clusters 3 and 6 were from PTC patients with concurrent HT. Other clusters were contributed by PTC patients without concurrent HT (Figure 3B). Clusters in samples from PTC patients without concurrent HT exhibited higher expression levels of TG (Figure 3C). CCDC80 was restrictedly expressed in follicular epithelial cells in samples from PTC patients with concurrent HT (Figure 3A). It has been reported that in human thyroid carcinoma, CCDC80 played the role as a putative tumor suppressor gene (Ferraro et al., 2013). CCDC80 could positively regulate E-cadherin expression by counteracting the negative regulation of zinc finger protein. Thus, the expression of CCDC80 prevents cancer progression such as EMT. CD55 was found to uniquely expressed in cluster 5 (Supplementary Figure 2D), which comprised follicular epithelial cells_JUN high, follicular epithelial cells_NEAT1 high, and follicular epithelial cells_TG high in samples from PTC patients without concurrent HT. CD55 was known as receptor of virus and bacterial and was detected in PTC (Lucas et al., 1996). Previous study has unveiled the role of CD55 to promote the adhesion of medullary thyroid carcinoma cells for rapid spread (Mustafa et al., 2004). Furthermore, the expression of TPO was restricted in follicular epithelial cells_TFF3 high in samples from PTC patients without concurrent HT (Supplementary Figure 2D). TFF3 was restrictedly expressed in follicular epithelial cells in samples from PTC patients without concurrent HT (Figure 3A). TFF3 has been studied as a promoter of EMT and tumor invasion in PTC (Lin et al., 2018). Enriched expression of TFF3 suggested a stronger invasion ability of cluster 4, which may contribute to LNM in PTC patients without concurrent HT.

Next, we distinguished DEGs of follicular epithelial cells between two groups of patients (Figure 3D). Follicular epithelial cells in samples from PTC patients without concurrent HT were observed to highly express FOS, JUN, TG, TPO, DUSP1, IER2, EGR2, and BTG2 (Figure 3D and Supplementary Figures 2B–D). Associated with low expression levels of ZFP36, those coregulated genes were believed to form a positive network in tumor cell proliferation (Canzoneri et al., 2020). FOS and JUN belong to the AP-1 transcription factor (TF) family; their ability was to form heterodimer and homodimer gene regulation network that balanced the progression of malignant follicular epithelial cells (Hess et al., 2004). We further calculated the TF expression patterns and enriched critical regulators of follicular epithelial cells using a reported method based on Microwell-seq data (Suo et al., 2018; Figure 3E). Binary treated heatmaps presented more complicated TF regulon in samples from PTC patients with concurrent HT (Supplementary Figures 3A,B). In samples from PTC patients without concurrent HT, we identified TFCP2L1 and IRF3 as the specific regulons in follicular epithelial cells_TFF3 high. Those regulons were known to play essential roles in tumor cell progression (Kotarba et al., 2018; Arwert et al., 2020). In samples from PTC patients with concurrent HT, RXRG was identified as the most specific regulon in follicular epithelial cells_CCDC80 high. RXRG was found to have positive correlation with better cancer prognostic (Joseph et al., 2019). Gene ontology enrichment of follicular epithelial cells from PTC patients without concurrent HT included oxidative stress response, protein folding, thyroid hormone metabolic process, and detoxification (Figure 3F). By contrast, samples from PTC patients with concurrent HT showed significantly high expression levels of immunoglobulin genes and interferon-stimulated genes such as IFI6 and IFI27 (Cheriyath et al., 2011). The expression of IFI27 was involved in cancer epithelial cell proliferation (Suomela et al., 2004). In the opposite way, the function of CCDC80 and lymphocyte infiltration could counteract malignancy progression of follicular epithelial cells in samples from PTC patients with concurrent HT. Interestingly, gene function analysis of specifically expressed genes in samples from PTC patients with HT contained humoral immune response, lymphocyte-mediated immunity, and adaptive immune response based on B cell activation (Figure 3G). Those immune responses suggested a potential cell network between follicular epithelial cells, infiltrating B cells, and myeloid cells in HT microenvironment that could affect tumor progression.

We next adopted a machine learning model named metric learning to verify the distinct molecular characteristics in follicular epithelial cells (de Vazelhes et al., 2020). We aimed to identify gene modules that contributed to the epithelial cell characteristics in samples from PTC patients with or without concurrent HT. In this case, a binary classifier was designed to predict the source of follicular epithelial cells in scRNA-seq data. We first generated training datasets and validation datasets by randomly picking expression matrix from follicular epithelial cells annotated in scRNA-seq data. The accuracy of predicting increased with the increase of the iterations (Supplementary Figure 2E). The final predicting classification was visualized using *t*-distributed stochastic neighbor embedding plot (PTC_HT: samples from PTC patients with concurrent HT, PTC: samples from PTC patients without concurrent HT; circles represent training datasets, cross represent validation datasets; Figure 3H). Follicular epithelial cells had barely no crossover between two classification labels. Training datasets and validation datasets showed a fairly fusion state (Supplementary Figure 2F). Heatmap demonstrated genes that contributed to the prediction (Figure 3J). Notably, we found that TG, FOS, and MALAT1 contributed greatly to the classification of samples from PTC patients without concurrent HT. The expression pattern of MALAT1 was validated in the TCGA-THCA cohort (Figure 3I). As a long non-coding RNA, recent studies have revealed the role of MALAT1 in cancer metastasis and PTC malignant process *via* several mechanisms such as angiogenesis and gene expression regulation (Huang et al., 2016). The metric learning results suggested MALAT1 as a potential biomarker to evaluate the malignancy of PTC in diagnosis.

### Profiling of Tumor-Infiltrating Immune Cells in Papillary Thyroid Cancer

**FIGURE 4:**
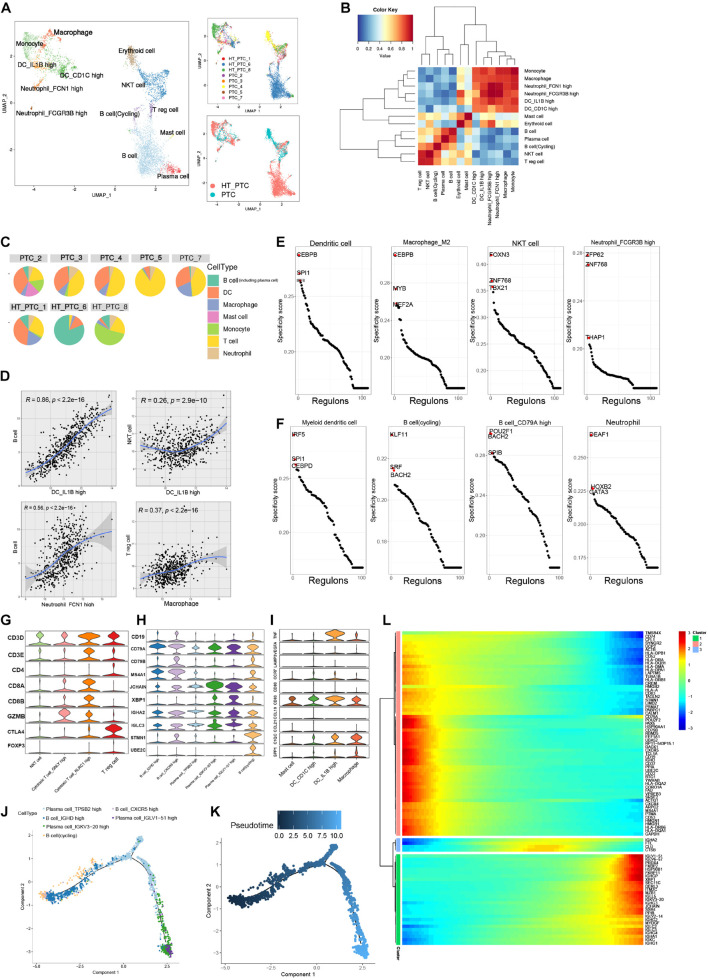
Reclustering of tumor-infiltrating immune cells. **(A)** UMAP plots of immune cell subclusters in PTC colored by cell types, patient, and PTC types. **(B)** Cell–cell Spearman correlation network of immune cell subclusters. **(C)** Pie charts of cell-type fractions for each patient’s tumor-infiltrating immune cells. **(D)** Correlation between myeloid cells and lymphocytes subsets in the TCGA-THCA cohort. Coefficient was calculated with Spearman correlation analysis. **(E)** Rank of the cell type–specific regulons in immune cells from PTC patients without concurrent HT. **(F)** Rank of the cell type specific regulon in immune cells from PTC patients with concurrent HT. **(G)** Violin plot of marker genes in T-cell subclusters. **(H)** Violin plot of marker genes in B-cell subclusters. **(I)** Violin plot of marker genes in myeloid cell subclusters. **(J)** Pseudotime trajectory of B-cell subclusters using monocle2. **(K)** Color-coded pseudotime of B-cell subclusters; the start point is dark blue. **(L)** Heatmap of the top DEGs expression levels in three divided clusters from pseudotime trajectory.

Reclustering of all tumor-infiltrating immune cells in tumor samples from PTC patients generated 13 tumor-associated immune cell subsets (Figure 4A). Common tumor-infiltrating lymphocytes consisted of NKT cells, regulatory T (Treg) cells, and follicular B cells, whereas most plasma cells and B cells were contributed by tumor samples from PTC patients with concurrent HT. The myeloid clusters included two DC cell subsets, two neutrophils subsets, macrophages, mast cells, and monocytes. A certain degree of immune cell composition variation could be observed between PTC types rather than patients, but a high proportion of lymphocytes could be observed in most patients (Figure 4C). Single cells were assigned into annotated clusters to calculate the Spearman correlation between lymphocytes and myeloid cells, respectively, (Figure 4B).

Significant enrichment of TF regulon activity could be observed in myeloid cells from PTC patients without concurrent HT. SPI1, ETS2, and CCAAT enhancer binding protein heterodimers (CEBPB, CEBPA, CEBPD, and CEBPG) were enriched in macrophages, neutrophils, DCs, and endothelial cells. Those activation patterns of myeloid cell were believed to participate in the regulation of tumor cell progression (Pitarresi et al., 2016; Figure 4E and Supplementary Figure 3A). In tumor-infiltrating B cells from PTC patients with HT, we identified POU2F1 and BATH2 as key regulons to maintain B cell identity (Miura et al., 2018; Pankratova et al., 2018; Figure 4F).

Papillary thyroid cancer infiltrating T cells consisted of CD3^+^ cytotoxic T cell, CD8^+^ cytotoxic cell, and CTLA4^+^ and CD4^+^ Treg cells (Figure 4G). Plasma cells were characterized by the prominent expression levels of JCHAIN and XBP1. Cycling B cells expressed dogmatic cell cycle genes such as STMN1 and UBE2C (Figure 4H). DC subsets expressed low levels of migration marker C-C motif chemokine receptor 7, but interleukin 1B^+^ (IL1B^+^) DCs expressed high levels of proinflammatory mediator genes such as tumor necrosis factor (TNF) and IL1B (Brown et al., 2019; Figure 4I). We observed positively strong gene expression signature correlations between IL1B^+^ DCs and follicular B cells in the TCGA-THCA cohort (Figure 4D), indicating that IL1B^+^ DCs could recruit B cells into TME in PTC patients. Thus, we performed trajectory analysis to investigate the B cell–infiltrating process (Figure 4J). Cycling B cells, B cells_IGHD high, and B cells_CXCR5 high were located in the start point of the pseudotime trajectory, whereas three subsets of plasma cells were enriched at the two branches (Figure 4K). Along with the immune responses caused by HT, infiltrating B cells proliferated and differentiated into plasma cells to generate thyroid-restricted antibodies. In this differentiation lineage, pseudotemporal expression heatmap showed scaled expression of dynamic genes (Figure 4L). Cluster 1 enriched the cycling B cell marker. Function analysis of enriched genes suggested B cell proliferation, differentiation, and leukocyte chemotaxis. Cluster 3 contains up-regulated genes related to the secreting of autoantibodies. Gene function enrichment analysis indicated immunoglobulin receptor binding, antigen binding, and Fc-gamma receptor signaling pathway.

### Distinct Molecular Signatures of Papillary Thyroid Cancer With or Without Concurrent Hashimoto’s Thyroiditis

**FIGURE 5:**
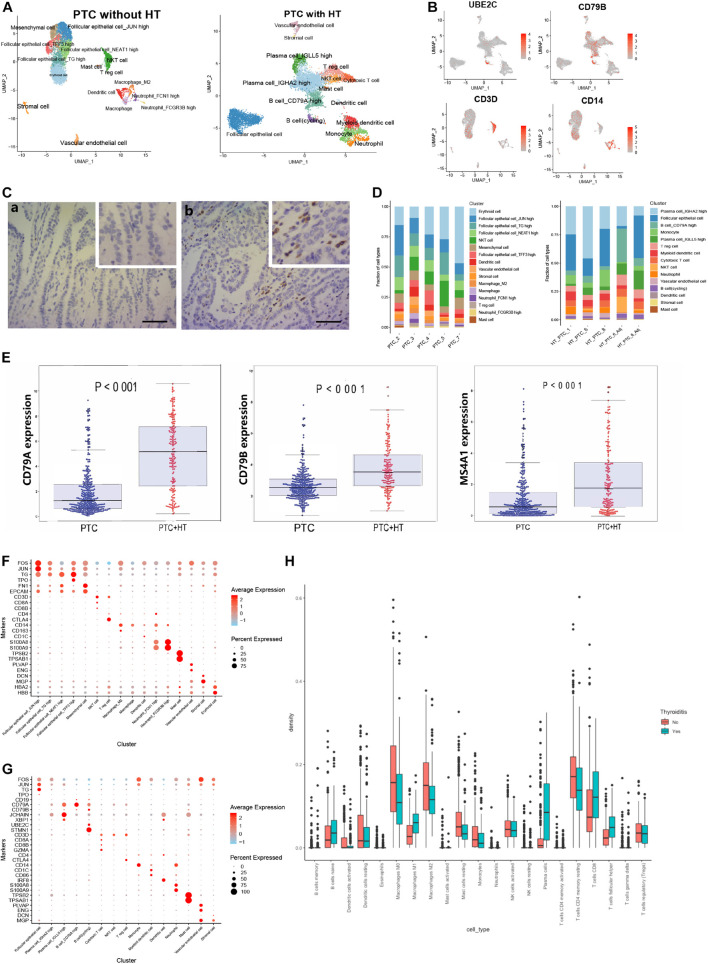
Reclustering of single cells in samples from two types of PTC. **(A)** UMAP plot of single cells in samples from two types of PTC. **(B)** FeaturePlot of selected marker genes in two types of PTC. **(C)** Immunostaining of CD79A/B in two types of samples from PTC patients. Positive signals were stained in brown. Scale bar = 50 μm. (a: sample from PTC patient without concurrent HT, b: sample from PTC patient with concurrent HT). **(D)** The fractions of cell types in samples from two types of PTC. **(E)** The expression levels of B cell marker genes in the TCGA-THCA cohort based on PTC types. PTC, PTC patients without concurrent HT; PTC + HT, PTC patients with concurrent HT. **(F)** Dot plot of marker genes in samples from PTC patients without concurrent HT. Average expression is color-coded. Columns represent the selected marker genes. **(G)** Dot plot of marker genes in samples from PTC patients with concurrent HT. Average expression is color-coded. Columns represent the selected marker genes. **(H)** Density of canonical immune cells in the TCGA-THCA cohort. Thyroiditis Yes, PTC patients with concurrent HT; Thyroiditis No, PTC patients without concurrent HT.

As considerable cell heterogeneity was observed between samples from PTC patients with or without concurrent HT, we reclustered cells based on patient type. The clusters in samples from PTC patients without concurrent HT mainly comprised four malignant follicular epithelial cell subsets, NKT cells, Treg cells, mesenchymal cells, DCs, macrophages, neutrophils, and mast cells (Figures 5A,B and Supplementary Figures 4A,C). Clusters in samples from PTC patients with concurrent HT comprised two plasma cell subsets, B cells, monocytes, NKT cells, myeloid DCs, cytotoxic T cells, Treg cells, follicular epithelial cells, and a small number of mast cells (Figures 5A,B and Supplementary Figure 4B). As a canonical B cell marker gene, CD79 was validated by IHC staining in samples from PTC patients with concurrent HT (Figure 5C). JCHAIN and XBP1 were enriched in plasma cells_IGLL5 high, plasma cells_IGHA2 high, B cells_CD79A high, and cycling B cells (Supplementary Figure 4D).

We next examined the cell compositions in two types of patients. Different patients barely showed bias in each cell type (Figure 5D). Cell fractions in samples from PTC patients without concurrent HT showed dominated status of follicular epithelial cells, which have been characterized by high expression of TG, JUN, FOS, and EPCAM (Supplementary Figures 5A–C). Mesenchymal cells and stromal cells were defined by FN1 and DCN, respectively. Neutrophils expressed high levels of S100A8 and S100A9. Mast cells specifically expressed TPSB2 and TPSAB1 (Figure 5F). Samples from PTC patients with HT included four plasma cell and B cell clusters (high expression of immunoglobulin, JCHAIN, XBP1, CD79A, and CD79B). In addition, cycling B cells were defined by UBE2C and STMN1 (Figure 5G). Monocyte was defined as high expression of CD14 molecule (CD14). DCs were identified using IRF8, CD1C, and CD86. Vascular endothelial cells showed high expression levels of ENG and PLVAP. Staining intensities of CD55 on samples from PTC patients without concurrent HT also showed relatively higher expression levels (Supplementary Figure 5D). To further investigate the patterns of infiltrating B cells in PTC patients with concurrent HT in the clinical datasets, PTC samples in the TCGA-THCA cohort were divided into two groups. We noticed B cell–related markers such as CD79A, CD79B, and MS4A1 were significantly enriched in PTC patients with concurrent HT (Figure 5E). Strikingly, tumor-infiltrating immune cell abundance profile of patients in the TCGA-THCA cohort also indicated significant enrichment of plasma cells and naive B cells in PTC patients with concurrent HT (Figure 5H).

### Tumor-Infiltrating B Lymphocytes With Migratory Capacity Unveil the Immunological Link Between Papillary Thyroid Cancer and Hashimoto’s Thyroiditis

**FIGURE 6:**
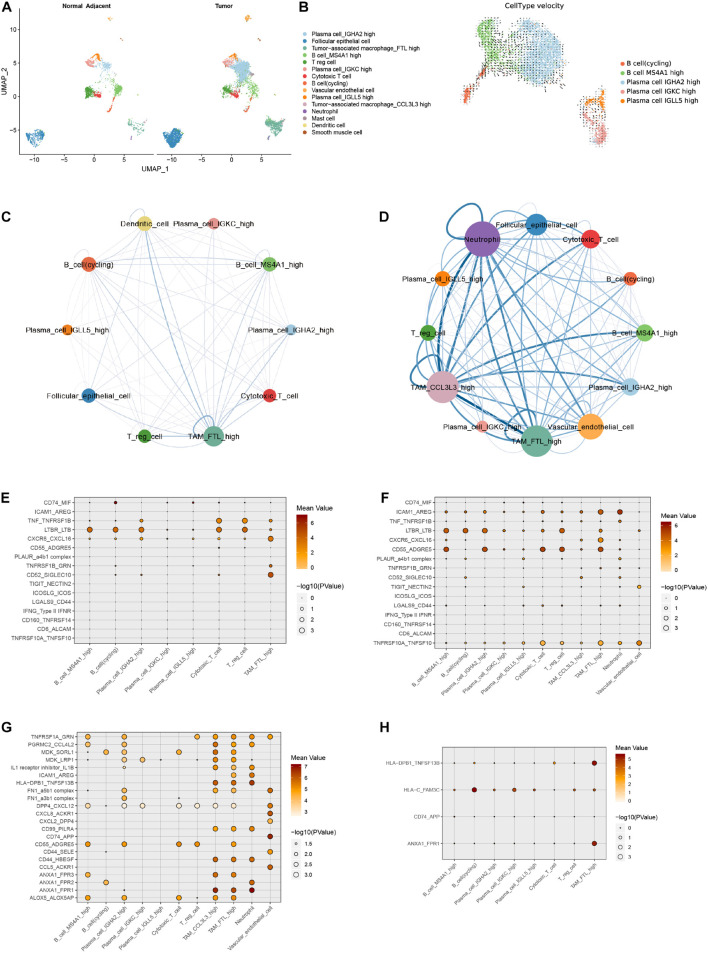
Comparison of adjacent tissues and tumor tissues from PTC patients with concurrent HT. **(A)** UMAP plot of cell types in adjacent tissues and tumor tissues from PTC patients with concurrent HT. **(B)** UMAP plot shows RNA velocities of B cell subsets in adjacent tissues and tumor tissues. **(C)** Cell–cell interaction network of ligand–receptor pairs in adjacent tissues of PTC patients with concurrent HT. The size of the circle represents the total ligand–receptor pairs of each cell type. The line weight represents the ligand–receptor pairs between two linked cell types. **(D)** Cell–cell interaction network of ligand–receptor pairs in tumor tissues of PTC patients with concurrent HT. **(E)** Selected ligand–receptor pairs of follicular epithelial cells from adjacent tissue samples in PTC patients with concurrent HT. *x* axis, receptor cells; *y* axis, ligand–receptor pairs (expression level is color-coded). **(F)** Selected ligand–receptor pairs of follicular epithelial cells from tumor samples in PTC patients with concurrent HT. **(G)** Calculated ligand–receptor pairs of follicular epithelial cells from adjacent tissue samples in PTC patients with concurrent HT. **(H)** Calculated ligand–receptor pairs of follicular epithelial cells from tumor samples in PTC patients with concurrent HT.

It is still not clear whether the infiltrating immune cell phenotypes in samples from PTC patients with concurrent HT were affected by adjacent HT microenvironment. We merge the single cells in tumor samples and adjacent tissue samples from PTC patients with concurrent HT (Figure 6A and Supplementary Figure 6A). Adjacent tissues from PTC patients with concurrent HT occupied the majority of cycling B cells, Treg cells, cytotoxic T cells, and MS4A1^+^ B cells (Supplementary Figures 6B,C). In contrast, tumor samples from PTC patients with concurrent HT occupied the majority of TAMs and IGHA2^+^ plasma cells. To delineate the path of B cell–to–plasma cell differentiation, we reclustered B cell subsets in tumor tissues and paired adjacent tissues from PTC patients with concurrent HT (Supplementary Figures 6D,E). RNA velocity analysis predicted the migration potential of B cells as well as cycling B cells and revealed their activity to infiltrate tumor tissues (Figure 6B and Supplementary Figure 6C). Considering the crucial role of myeloid cells in lymphocyte recruitment (Ehlers and Schott, 2014), we investigated the cell–cell ligand–receptor networks in tumor samples and paired adjacent tissues from PTC patients with concurrent HT, respectively. Adjacent tissue showed much fewer ligand–receptor pairs compared to tumor samples (Figures 6C,D). TAMs, neutrophils, and DCs enriched most ligand–receptor pairs with lymphocytes. These findings highlight myeloid cells as a potential signal transition hub to regulate the B cell recruitment from adjacent tissues to tumor tissues.

We adopted CellphoneDB to detect common ligand–receptor interactions in thyroid cancer that have been alternately verified in other cancers recently (Chen et al., 2020; Lee et al., 2020). Dot plots showed selected ligand–receptor interactions starting from follicular epithelial cells to immune cells in adjacent tissue and tumor samples from PTC patients with concurrent HT, respectively, (Figures 6E,F). These cell–cell interaction networks indicated more positive interactions between follicular epithelial cells and immune cells in tumor samples. Protumor interactions such as TNF_TNFRSF1B, LTBR-LTB, and CXCR6_CXCL16 were found to have higher expression levels in follicular epithelial cells. LTBR interacted with lymphocyte LTB and promoted tumor cell metastasis (Das et al., 2019). Transmembranous CXCL16 and soluble CXCL16 played opposite roles in tumor cell migration (Gooden et al., 2014). Another pair TNF_TNFRSF1B could activate the nuclear factor κB pathway and resisted TNF-induced tumor cell death (Rothe et al., 1995). Those previously reported mechanisms in other cancers seem to have the synchronization regulation pattern in PTC with concurrent HT. Follicular epithelial cells in tumor samples from PTC patients with concurrent HT exclusively contained the antitumor interacting pair TNFRSF10A_TNFSF10. TNFSF10 has been reported to induce tumor cell apoptosis (Qu et al., 2019). These potential antitumor interaction networks were constructed by plasma cells, cytotoxic T cells, TAMs, and vascular endothelial cells.

We then calculated other notable interaction pairs of epithelial cells in tumor tissues and adjacent tissues from PTC patients with concurrent HT (Figures 6G,H). Interacting pairs HLA-DPB1_TNFSF13B, HLA-C_FAM3C, and ANXA1_FPR1 were observed between follicular epithelial cells and macrophages. TNFSF13B has been recognized as a potent B cell activator cytokine (Nocturne and Mariette, 2015). FAM3C and FPR1 were reported to be related to malignant cell migration (Cao and Zhang, 2018; Yin et al., 2018). In adjacent tissues, the myeloid cell–mediated lymphocyte activation may form an immune response network to resist PTC progress. In follicular epithelial cells from tumor samples, cytokine interactions were observed in ACKR1-positive endothelial cells. Endothelial cells that highly expressed ACKR1 and PLVAP could enhance the transmigration of lymphocytes (Ramachandran et al., 2019), and GRN was found to promote angiogenesis and tumor cell proliferation (Yang et al., 2015). Ligand–receptor pairs of TAMs_FTL^+^ in samples from PTC patients with concurrent HT contained quite a number of cytokines (Supplementary Figures 7A,B). Tumor-associated macrophage_CCL3L3 and neutrophils in samples from PTC patients with concurrent HT showed similar patterns (Supplementary Figures 7C,D). In fact, a fair proportion of interaction pairs including cytokines and major histocompatibility complex could somehow facilitate the progression of PTC (Miyake et al., 2016). Conversely, lymphocyte infiltration patterns induced by endothelial cells could inhibit the growth and migration of PTC *via* boosting the effect of T cells and improve clinical prognostic (Wouters and Nelson, 2018).

## Discussion

Tumor heterogeneous and immune microenvironments have been considered to significantly impact the development of cancer. Different cell subsets in tumors could regulate this process with varying degrees. Conventional bulk RNA-seq of tumors vaguely described the gene expression modules at tissue level. In this work, we generated the single-cell transcriptome landscape of human PTC. The PTC landscape covered 24 individual cell clusters from eight PTC patients, providing a rich resource for multidimensional characterization of parenchymal cells and immune cells in PTC. Inferred CNVs in parenchymal cells identified predicted tumor cells in follicular epithelial cells and mesenchymal cells with strong EMT properties, which may lead to tumor progression and metastasis. The *BRAF* V600E mutation confers an aggressive phenotype in PTC, and a previous study has used RNA-seq to identify DEGs between *BRAF* V600E mutation and *BRAF* wild-type tumors (Smallridge et al., 2014). Nonetheless, single-cell profiling revealed individual tumor cell characteristics that may be obscured in bulk analysis. Our single-cell analysis revealed that *BRAF* V600E mutation tumor epithelial cells highly expressed TACSTD2 and CLDN3, which were then validated in TCGA data. It was intriguing that analysis of TCGA data indicates a strong correlation between TACSTD2, CLDN3, and DFS in PTC, implying that *BRAF* V600E mutation might be related to TACSTD2 through ERK and JNK pathways and the upregulation of MMP2. LNM in PTC has also been shown to be associated with locoregional recurrence and poor prognosis. Single-cell analysis identified that CTSC and B2M were up-regulated in LNM-positive epithelial cells with strong correlation to poor DFS, suggesting that cancer-related signaling pathways such as TNF-α/p38 MAPK signaling pathway could promote LNM in PTC (Zhang et al., 2020). Reclustering of follicular epithelial cells defined epithelial cells high expressed TFF3 in samples from PTC patients without concurrent HT and epithelial cells high expressed CCDC80 in samples from PTC patients with concurrent HT. Machine learning model predicted MALAT1 as a potential biomarker in PTC patients without concurrent HT.

The dynamic responses of immune system in PTC remain to be completely deciphered. We revealed the complexity of immune cells in PTC and identified multiple PTC-specific myeloid cells, T cells, and B cell subsets. In the TCGA-THCA cohort, single-cell gene expression signatures of proinflammatory IL1B^+^ DC cells were highly positively correlated with B cells. The proinflammatory DCs continually recruited lymphocytes into TME and suppressed T cell exhaustion. High proportion of tumor-infiltrating lymphocytes could explain the indolent properties of PTC, which leads to favorable prognosis. Intriguingly, we found B lymphocyte and plasma cell enrichment pattern in PTC tumor tissues that were concurrent with HT, but not in the PTC alone without concurrent HT. B lymphocyte infiltration in TME has been identified in multiple types of cancers such as lung cancer and was recognized as the main effector cells of humoral immunity, which suppress tumor progression (Wang et al., 2019). A great number of ligand–receptor interactions were observed between B cells, T cells, DCs, and TAMs. Precursor–progeny relationship supported by RNA velocity unveiled the migration potential of infiltrating B cells from adjacent tissues to tumor tissues. PTC is always accompanied by the synchronous appearance of HT. Compared with adjacent tissues, more complicated cell–cell interaction networks of ligands and receptors between endothelial cells, epithelial cells, lymphocytes, and myeloid cells in samples from PTC patients with concurrent HT suggested a controlled homeostasis microenvironment of tumor progress regulation. The analyses of PTC and adjacent HT tissues implied that the cellular TME was reshaped by the B lymphocytes derived from adjacent HT tissues. Based on our observations, we hypothesized that B lymphocyte–related immune response was a possible reason for the better prognosis of PTC patients with concurrent HT. But detailed mechanisms need further investigation and verification. Also, limited number of cases in our study could not cover all the clinical features of PTC, which remains to be further explored.

In summary, our work is a comprehensive systematic single-cell transcriptome survey of human primary PTC. We revealed detailed molecular characteristics of PTC cells as well as their clustering, dynamic, and developmental trajectory. Our analysis uncovered B cells infiltrating in tumor tissues as a distinctive feature for PTC patients with concurrent HT. Our findings are potentially valuable in not only serving as a resource for deeper understanding of PTC in general, but also elucidating the immunological correlation between PTC and HT.

## Data Availability Statement

The datasets presented in this study can be found in online repositories. The names of the repository/repositories and accession number(s) can be found below: https://www.ncbi.nlm.nih.gov/geo/, GSE163203.

## Ethics Statement

The studies involving human participants were reviewed and approved by Clinical Research Ethics Committee of The first affiliated hospital, school of medicine, Zhejiang university (IIT consent: NO.700,2020). The patients/participants provided their written informed consent to participate in this study.

## Author Contributions

YjW, GG, JP, and FY conceived and designed the experiments. JP, QZ, HT, MZ, and YZ collected the patients’ samples. JP, FY, YS, FZ, and XZ performed the experiments. FY, CY, YY, JL, and JP analyzed the data. FY, JP, and QZ wrote the manuscript. YnW, JP, and YjW provide funding and supervision. All authors read and approved the final manuscript.

## Conflict of Interest

The authors declare that the research was conducted in the absence of any commercial or financial relationships that could be construed as a potential conflict of interest.

## Publisher’s Note

All claims expressed in this article are solely those of the authors and do not necessarily represent those of their affiliated organizations, or those of the publisher, the editors and the reviewers. Any product that may be evaluated in this article, or claim that may be made by its manufacturer, is not guaranteed or endorsed by the publisher.

## Footnotes

1
http://mccarrolllab.com/wp-content/uploads/2016/03/Drop-seqAlignmentCookbookv1.2Jan2016.pdf

2
https://github.com/ggjlab/mca_data_analysis/tree/master/preprocessing

3
https://satijalab.org/seurat/

4
https://db.cngb.org/HCL/index.html

5
https://xena.ucsc.edu/

6
http://velocyto.org/velocyto.py/tutorial/index.html

7
https://github.com/KevinMusgrave/pytorch-metric-learning

8
https://captum.ai/

## Abbreviations

CNV: copy number variations
DCs: dendritic cells
DEGs: differentially expressed genes
DFS: disease-free survival
EMT: epithelial–mesenchymal transition
GSVA: gene set variation analysis
HT: Hashimoto thyroiditis
LNM: lymph node metastasis
NK: natural killer cells
NKT: natural killer T cells
PTC: papillary thyroid cancer
scRNA-seq: single-cell RNA sequencing
RFS: recurrence-free Survival
TF: transcription factor
TME: tumor microenvironment
Treg: regulatory T cells
UMAP: Uniform Manifold Approximation and Projection.
