# Supplementary material for: Papillary Thyroid Carcinoma Landscape and Its Immunological Link With Hashimoto Thyroiditis at Single-Cell Resolution
Source: Front Cell Dev Biol. 2021 Nov 5;9:758339. doi: 10.3389/fcell.2021.758339 (PMC8602800; doi:10.3389/fcell.2021.758339)

Supplementary figure.1

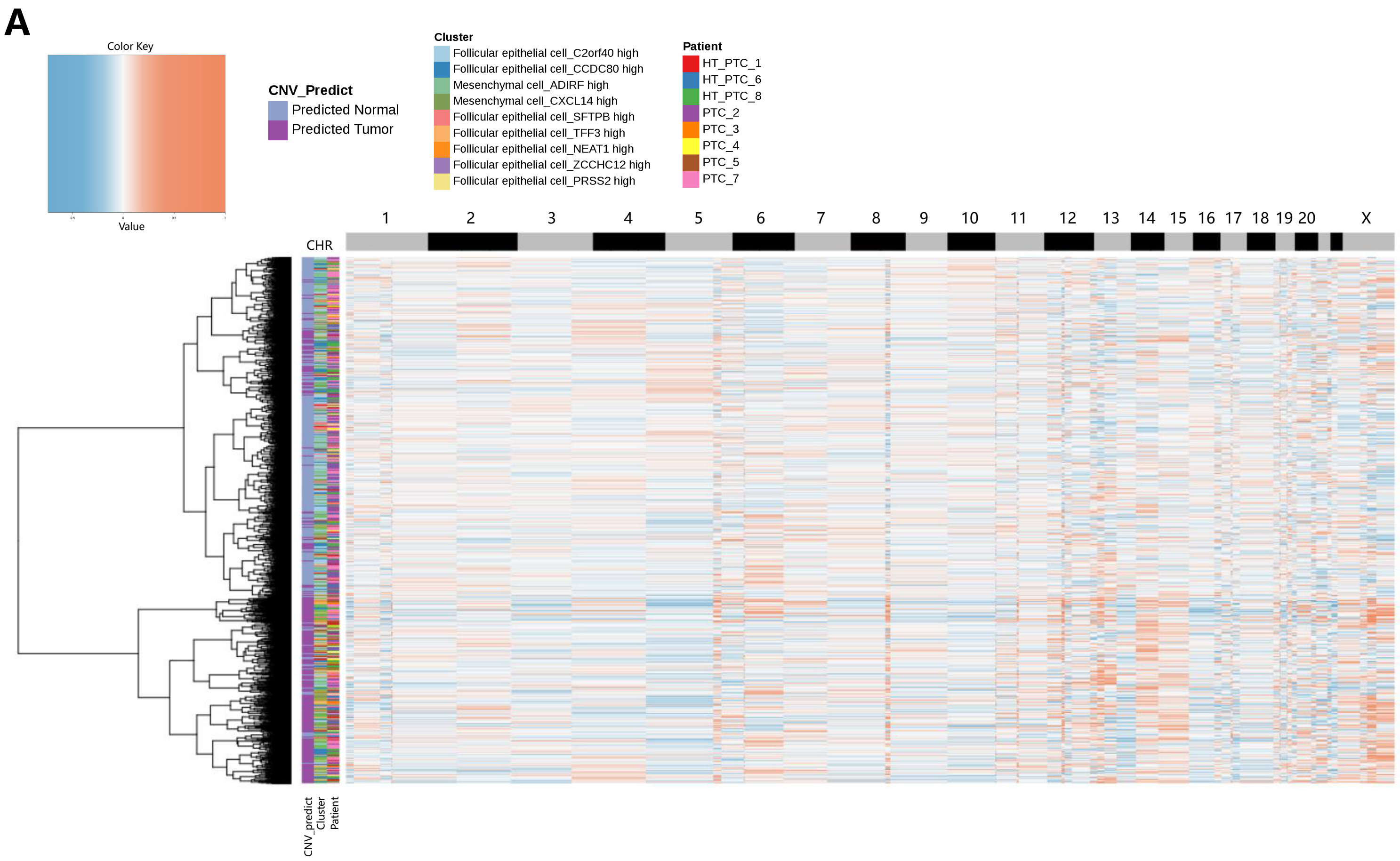

Supplementary figure.2

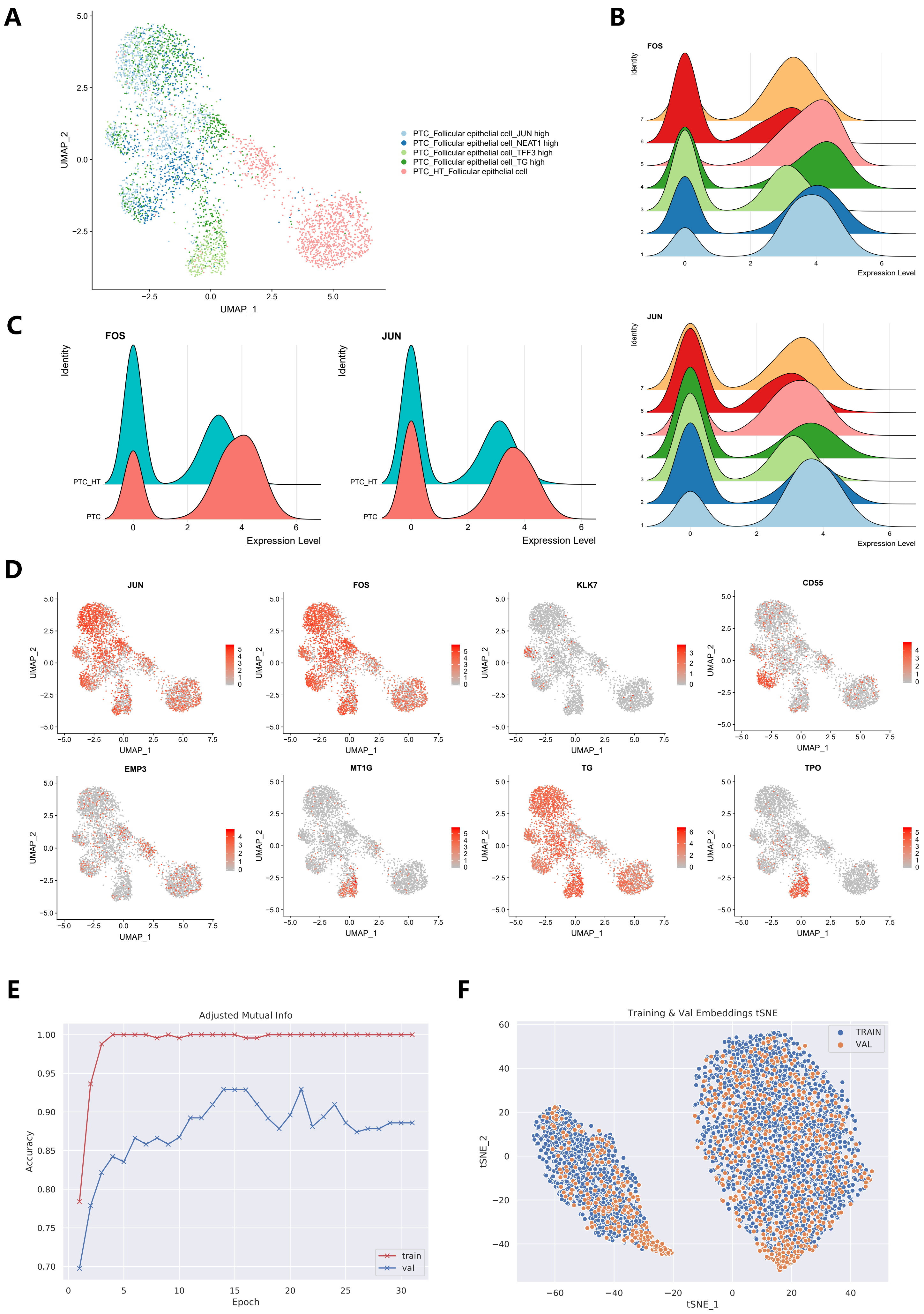

Supplementary figure.3

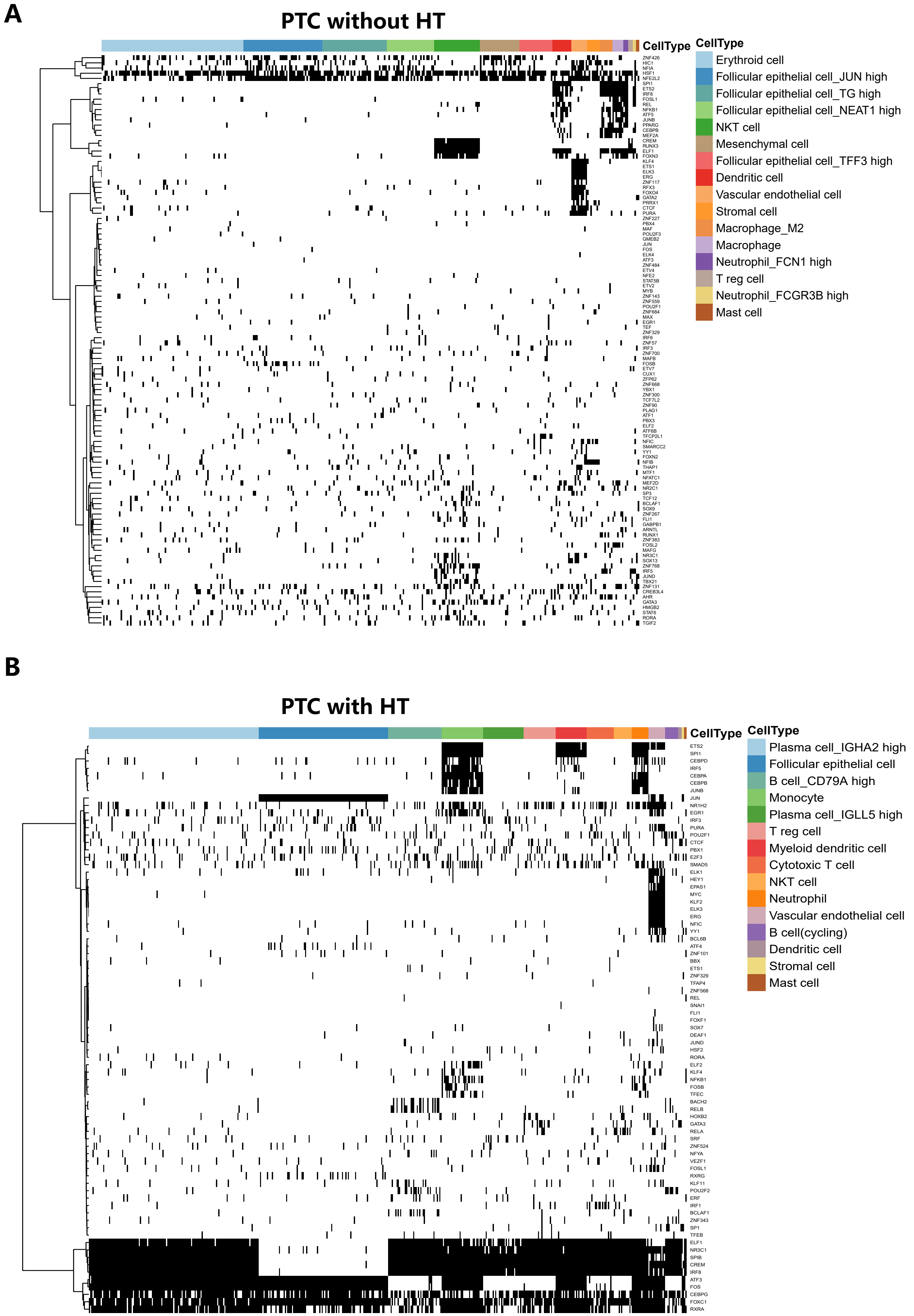

Supplementary figure.4

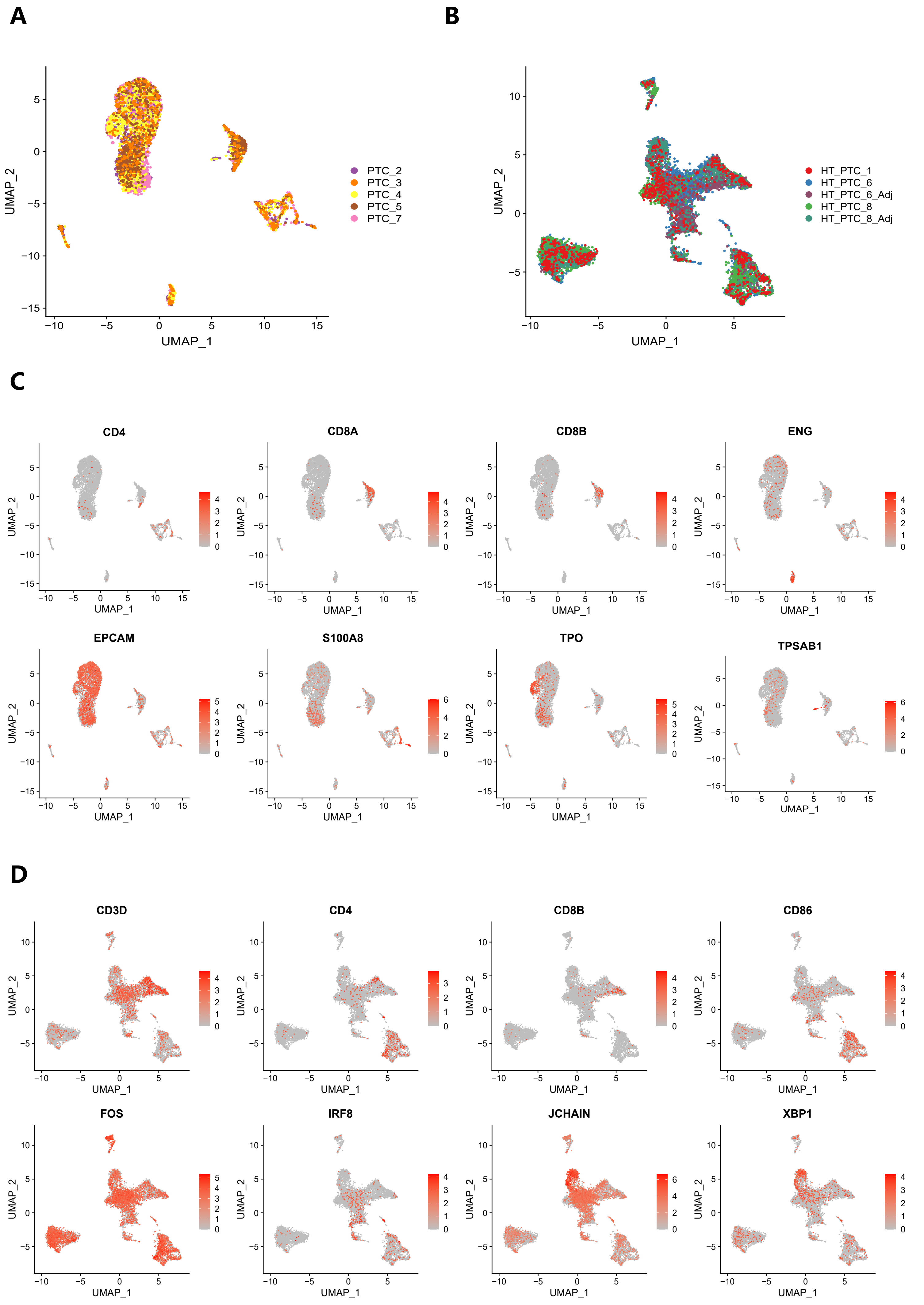

Supplementary figure.5

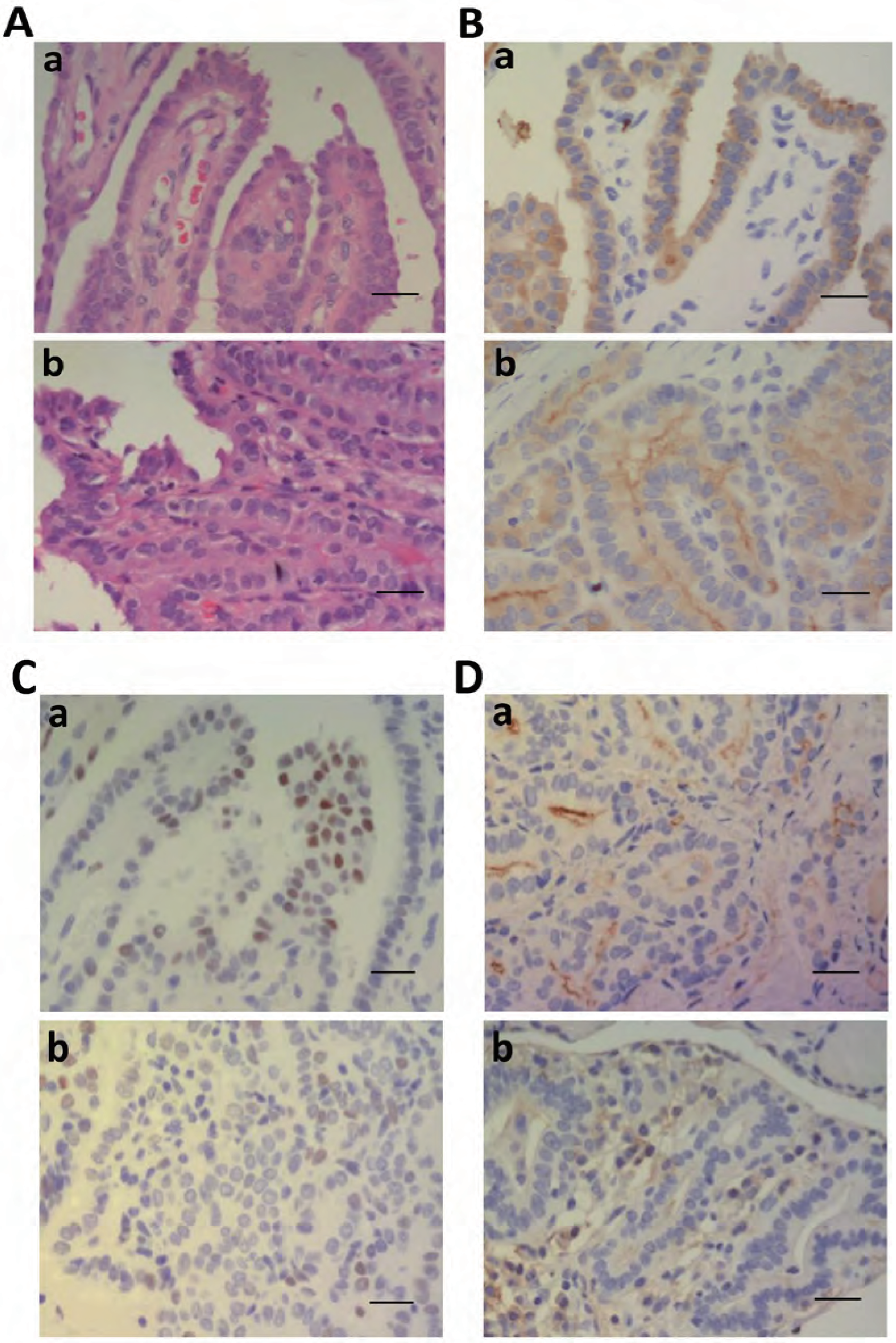

Supplementary figure.6

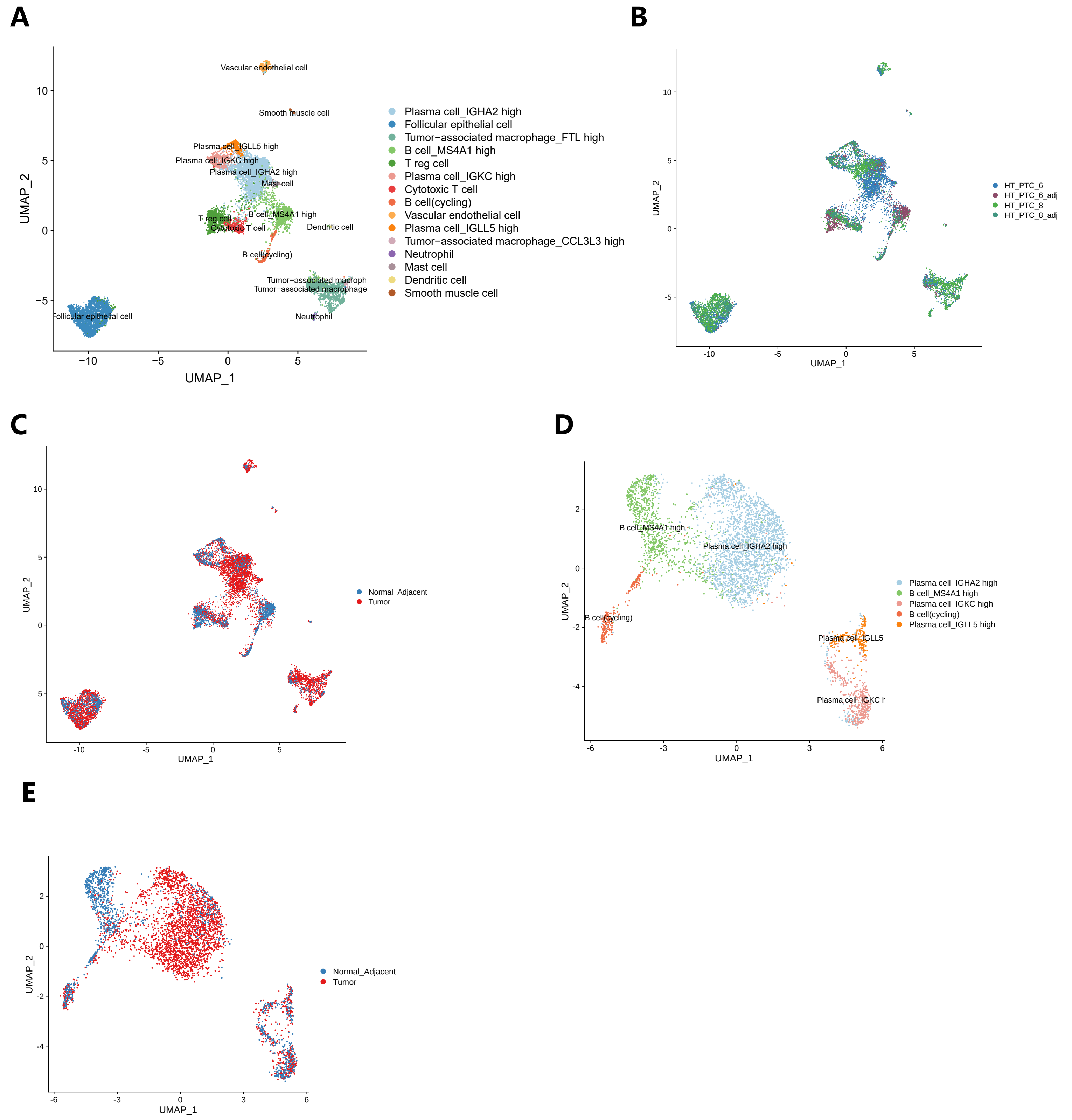

Supplementary figure.7

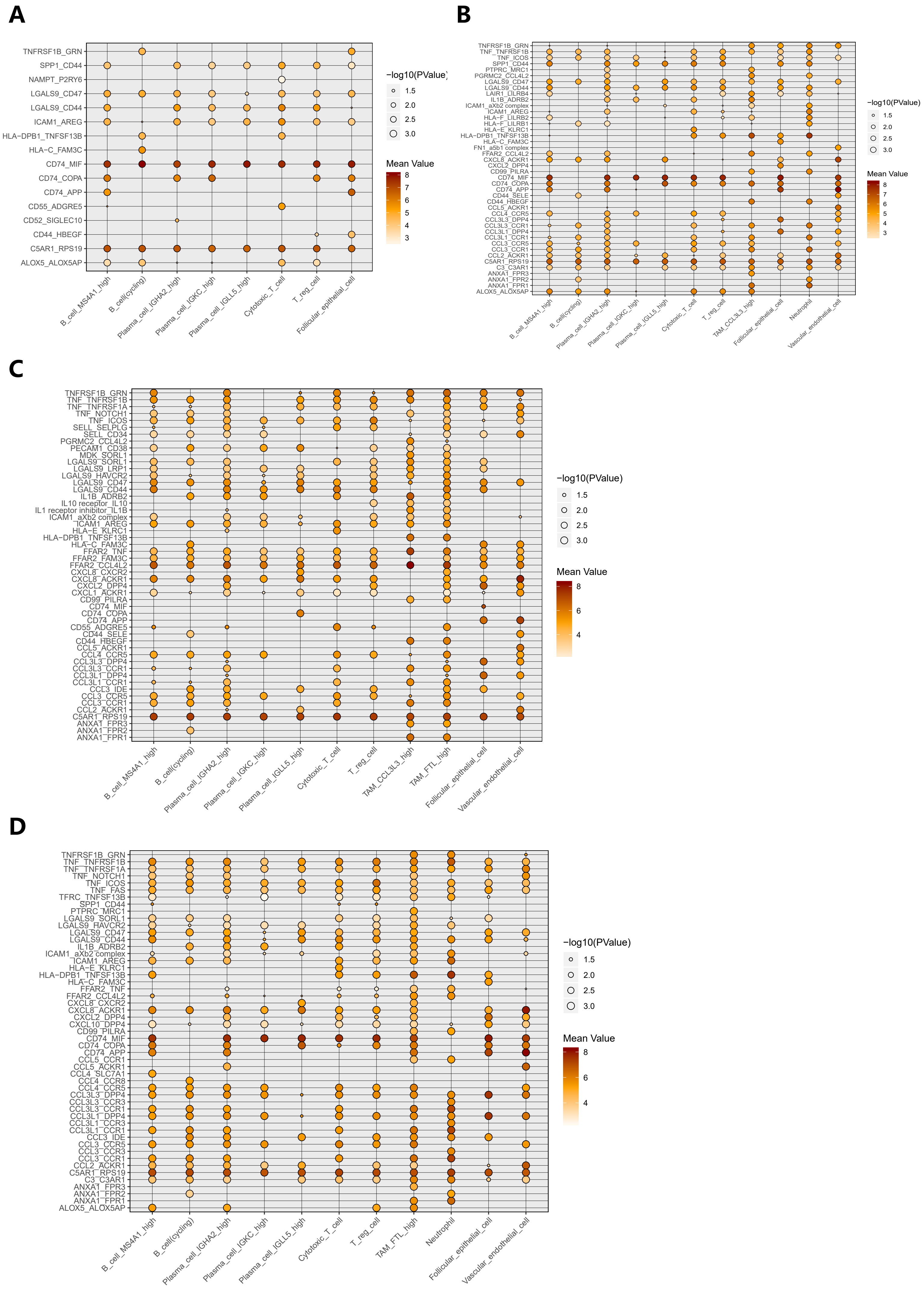

Supplement: Supplementary file 1 [file Data_Sheet_1.PDF]
