# Supplementary material for: Papillary Thyroid Carcinoma Landscape and Its Immunological Link With Hashimoto Thyroiditis at Single-Cell Resolution
Source: Front Cell Dev Biol. 2021 Nov 5;9:758339. doi: 10.3389/fcell.2021.758339 (PMC8602800; doi:10.3389/fcell.2021.758339)
Supplement: Supplementary file 2 [file Data_Sheet_2.docx]

**Supplementary Figure.1 Predicted copy number variations (CNVs) of parenchyma cells.**

1. Heatmap of predicted CNVs of parenchyma cellssubclusters.

**Supplementary Figure. 2 Comparison of follicular epithelial cells.**

1. UMAP plotshows the cell type original annotations of follicular epithelial cells.
2. RidgePlots of FOS and JUN expression levels and cell number distributions in follicular epithelial cells subclusters.
3. RidgePlots of FOS and JUN expression levels and cell number distributions in follicular epithelial cells from PTC patients with or without concurrent HT.
4. FeaturePlots of selected marker genes in follicular epithelial cells.
5. Training process of PyTorch Metric Learning. Epoch(X-axis) represent the rounds of train. Y-axis represent the training accuracy.
6. t-SNEplot showing the distribution of training sets and validation sets in metric learning model.

**Supplementary Fig. 3 Heatmap of transcription factor regulons in PTC.**

1. Heatmap shows the binary treated expression of transcription factors of samples from PTC patients without concurrentHT.
2. Heatmap shows the binary treated expression of transcription factors of samples from PTC patients with concurrentHT.

**Supplementary Fig. 4 Merged samples from different PTC patients and marker genes expression patterns. (PTC: PTC without concurrent HT, HT_PTC: PTC with concurrentHT, Adj:adjacent tissue of PTC)**

1. UMAP plot of samples from PTC patients without concurrentHT.
2. UMAP plot of samples from PTC patients with concurrentHT.
3. FeaturePlotsshow selected marker genes of samples from PTC patients without concurrentHT.
4. FeaturePlotsshow selected marker genes of samples from PTC patients with concurrentHT.

**Supplementary Fig. 5 Immunostaining of selected genes in two groups of PTC samples. (Scale bars=25μm. (a: PTC patient without concurrent HT, b: PTC patient with concurrentHT)**

1. HE staining of samples from PTC patient without concurrentHT and sample from PTC patient with concurrentHT.
2. Immunostaining ofTG in two groups of samples from PTC patients. Positive signals were stained in brown.
3. Immunostaining of JUN in two groups of samples from PTC patients. Positive signals were stained in brown.
4. Immunostaining of CD55 in two groups of samples from PTC patients. Positive signals were stained in brown.

**Supplementary Fig. 6 UMAP plots of adjacent tissuesand tumortissues from PTC patients with concurrentHT.**

1. UMAP plot shows the cell types in adjacent tissues and tumor tissues.
2. UMAP plot of merged clusters colored by patients.
3. UMAP plot of merged clusters colored by adjacent tissues and tumor tissues.
4. UMAP plot shows theB cells subsets of adjacent tissues and tumor tissues.
5. UMAP plot shows the B cells subsets colored by adjacent tissues and tumor tissues.

**Supplementary Fig.7. Ligand-receptor interactions of adjacent tissues and tumor tissues from PTC patients with concurrentHT****. (X-axis: receptor cells, y-axis: ligand_recetptor pairs, expression levelsare color-coded)**

1. Ligand-receptor pairs from tumor-associated macrophages_FTL+ in adjacent tissues from PTC patients withconcurrentHT.
2. Ligand-receptor pairs from tumor-associated macrophages_FTL+ in tumor tissues from PTC patients with concurrentHT.
3. Ligand-receptor pairs from neutrophils in tumor tissues from PTC patients with concurrentHT.
4. Ligand-receptorpairs fromtumor-associated-macrophages_CCL3L3+ in tumor tissues from PTC patients with concurrentHT.
